# Germline activating sequence variations in RASopathy spectrum genes: genotype–phenotype correlation in a North Indian cohort

**DOI:** 10.3389/fgene.2025.1677143

**Published:** 2025-11-11

**Authors:** Shifali Gupta, Priyanka Srivastava, Roshan Daniel, Chitra Bhardwaj, Parminder Kaur, Pratibha Bawa, Anu Kumari, Ravi Pratap Singh Bhadoriya, Mrinali Peters, Anupriya Kaur, Inusha Panigrahi

**Affiliations:** Genetic Metabolic Unit, Department of Pediatrics, Advanced Pediatrics Centre, Post Graduate Institute of Medical Education and Research, Chandigarh, India

**Keywords:** Rasopathy, germline variants, next-generation sequencing, genotype-phenotype correlation, drug gene interaction

## Abstract

**Background:**

RASopathies represent a group of genetically heterogeneous developmental disorders caused by germline variants in genes regulating the RAS/MAPK signalling pathway. These syndromes share overlapping clinical features, complicating diagnosis. Dysregulation of this pathway disrupts normal development and contributes to diverse phenotypic manifestations. In this study, we conducted a comprehensive clinico-genetic correlation analysis in a North Indian cohort to identify causative genetic variants. As an exploratory approach, we also applied bioinformatic tools to identify potential drug–gene interactions.

**Methods:**

We enrolled 84 patients with clinical suspicion of RASopathy spectrum disorders. Whole exome sequencing (WES) was performed to detect pathogenic variants. Clinical data were systematically collected and analysed for genotype–phenotype correlation. Potential therapeutic agents were queried using the Drug–Gene Interaction database (DGIdb).

**Results:**

Pathogenic or likely pathogenic variants in RASopathy-related genes were identified in 46 cases, including 14 with neurofibromatosis. Two patients had variants of unknown significance (VUS) in *MAPK1* and *LZTR1*. *PTPN11* variants were detected in 12 patients, while variants in *LZTR1, MAP2K2, BRAF, NRAS, HRAS, RAF1, RIT1, SOS1,* and *SOS2* were found in 20 others. All identified variants were heterozygous missense mutations, consistent with autosomal dominant inheritance. The most common clinical features were short stature (64.7%), downslanting palpebral fissures (38.23%), and chest wall deformities (35.29%). A trend was observed for the association between PTPN11 and SOS1 variants and pulmonary stenosis, though not statistically significant due to the small cohort size. Unique phenotypic findings associated with different variants in RASopathy spectrum genes were noted. Exploratory DGIdb analysis highlighted candidate drug–gene interactions that may inform future research directions.

**Conclusion:**

Our findings underscore the clinical and genetic diversity of RASopathies in the Indian population and highlight the role of next-generation sequencing in early and accurate diagnosis. While exploratory drug–gene interaction analysis provides hypothesis-generating insights, clinical translation requires rigorous validation in functional studies and clinical trials.

## 1 Introduction

RASopathy is a broad term that consists of a genetically heterogeneous group of conditions with overlapping features. It affects approximately 1/1000 human births via germ-line variations in RAS/MAPK pathway components, leading to developmental disorders (Rauen, 2013). It is the largest group of known malformation syndromes. Ras/MAPK pathway helps in cell cycle regulation, growth, differentiation and senescence, i.e., all vital steps of a cell. Disturbance in Ras/MAPK pathways leads to deleterious effects on embryonic as well as later stages of development, which results in genetic pleiotropy. Disorders of the RAS/MAPK pathway include Noonan syndrome (NS), cardio-facio-cutaneous (CFC) syndrome, LEOPARD syndrome, Costello syndrome, Neurofibromatosis type 1 (NF1), Legius syndrome, etc.

Pathogenic variants that enhance RAS/MAPK signalling can be broadly classified based on their functional impact. Gain-of-function (GoF) mutations typically occur in genes encoding components that activate the signalling cascade, such as *PTPN11/SHP2, SOS1, RAS* GTPases, and RAF kinases. In contrast, loss-of-function (LoF) or dominant-negative (DN) mutations tend to affect genes encoding inhibitory regulators of the pathway, including neurofibromin, *SPRED1, SPRED2*, and *LZTR1*. Collectively, these alterations lead to upregulated RAS/MAPK pathway activity (Tartaglia et al., 2022).

Each RASopathy disorder has unique features, but due to common mechanisms in the pathway, clinical features are quite overlapping, which include short stature, cardiovascular defects, characteristic facial features, and cutaneous findings. Additional features, such as intellectual disabilities, developmental delays, and risk of malignancies, can also be associated with the RASopathies. It is important from a clinical viewpoint to delineate the underlying genetic aetiology in these syndromes as the therapy and response to treatment vary (Hebron et al., 2022).

Current treatment strategies for RASopathies are primarily symptomatic and supportive, as no curative therapies are yet approved. However, targeted therapies are emerging, particularly MEK inhibitors (such as selumetinib), which have shown promise in clinical trials for certain manifestations, like inoperable plexiform neurofibromas in NF1 (Gross et al., 2020). Other investigational drugs targeting the RAS/MAPK pathway, including RAF and SHP2 inhibitors, are under evaluation (Romero et al., 2020). The Drug–Gene Interaction database (DGIdb) serves as a valuable resource for identifying potential drug–gene interactions relevant to RASopathies (Cotto et al., 2018; Freshour et al., 2021). By querying genes commonly mutated in these syndromes (e.g., *PTPN11, KRAS, BRAF, NF1*), researchers and clinicians can explore existing or repurposed drugs that may modulate the dysregulated signalling pathways, thereby opening new avenues for precision therapy. Our primary aim was to delineate the clinical and molecular findings spectrum of RASopathies in a North Indian patient cohort with RASopathies by combining detailed phenotyping with next-generation sequencing (NGS). In addition, we performed an exploratory analysis using the DGIdb to map potential drug–gene associations for the identified variants. This analysis was not intended to guide immediate clinical decisions but rather to highlight candidate interactions that could be explored in future functional or translational studies. By integrating molecular findings with clinical features, our study seeks to contribute to improved diagnosis, genetic counselling, and hypothesis generation for future precision medicine approaches in RASopathies.

## 2 Methodology

### 2.1 Patients

**FIGURE 1:**
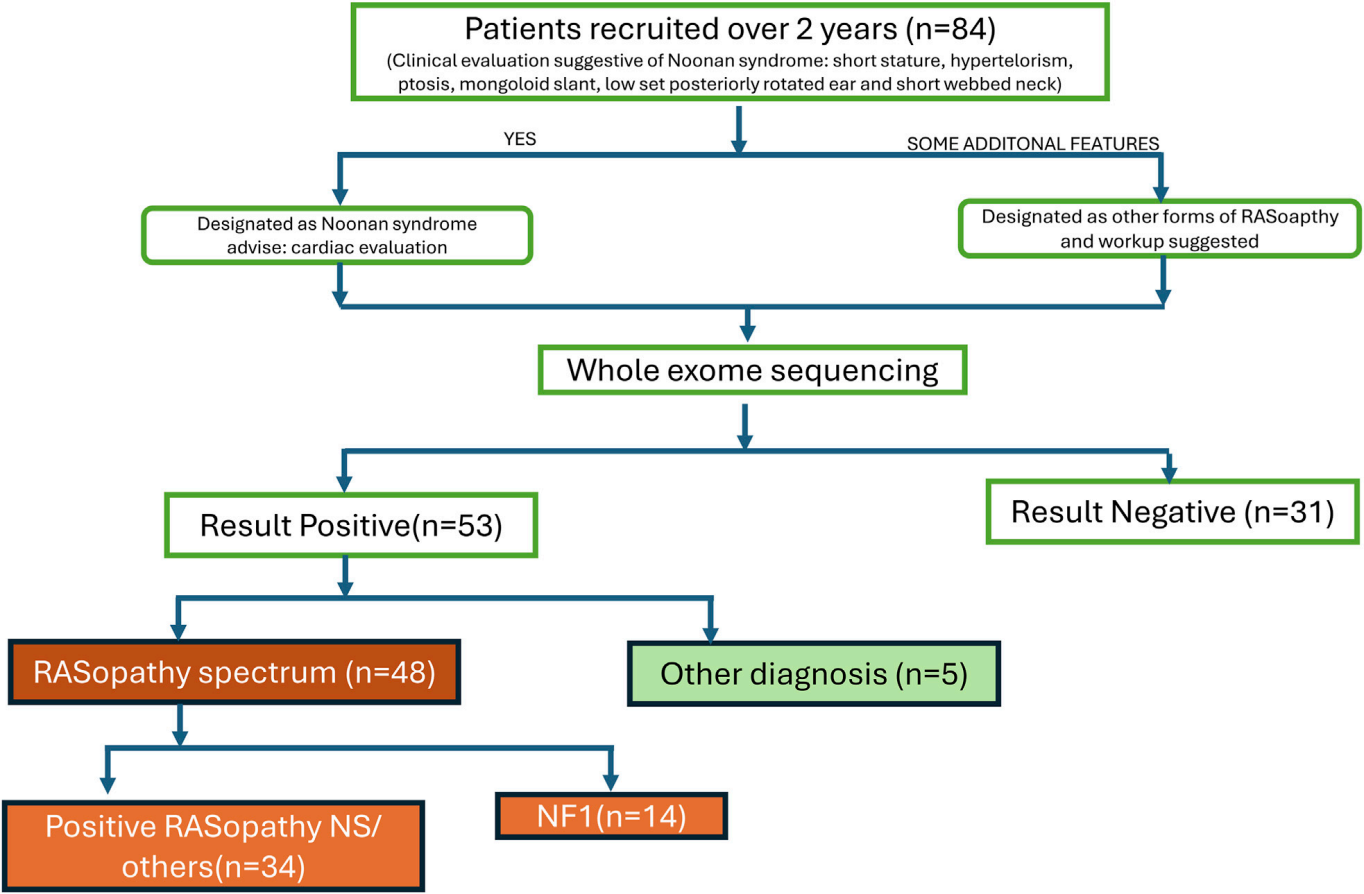
Workflow of the patients enrolled in the study.

**FIGURE 2:**
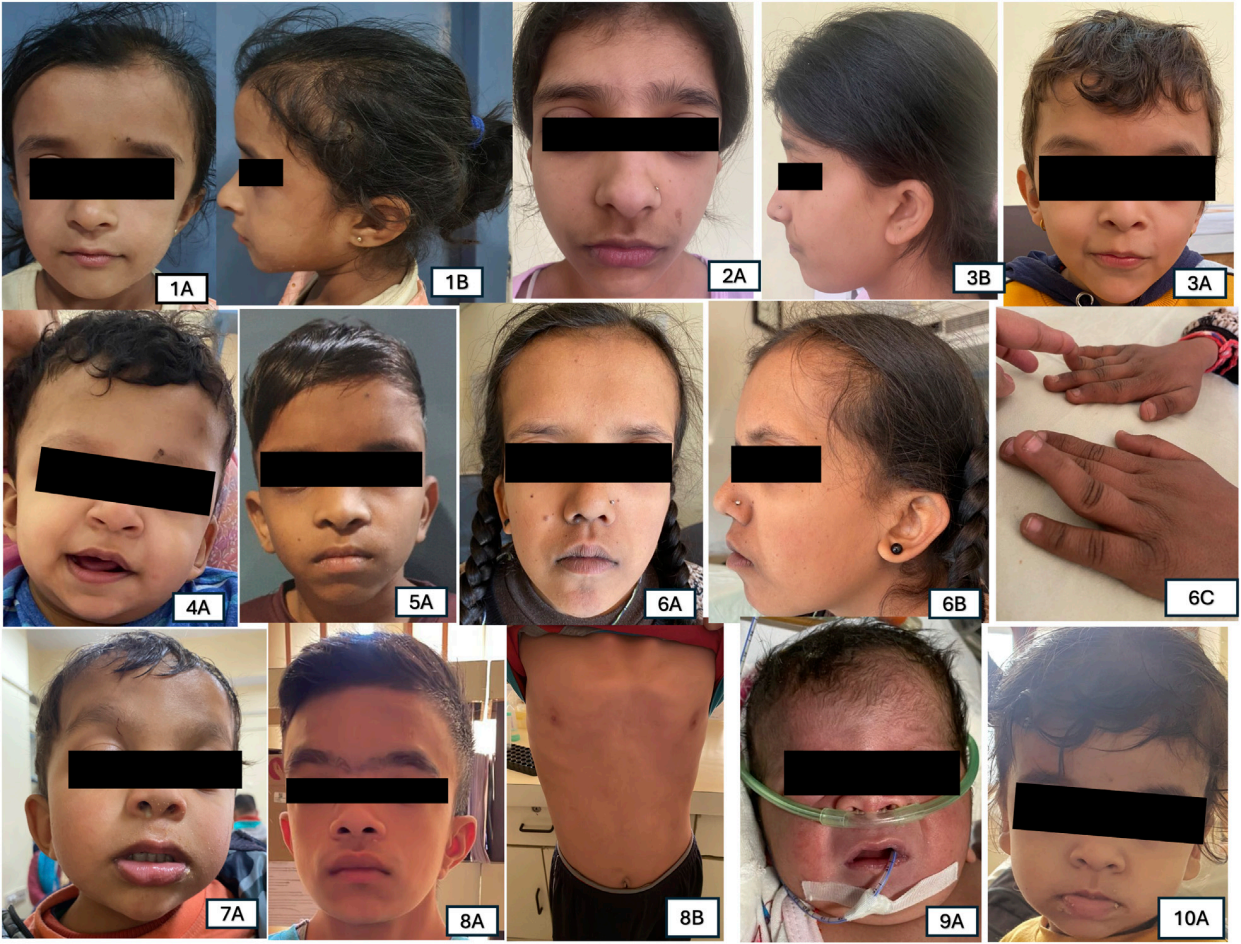
Representative pictures of Individuals with variants identified in various RASopathy genes. Patient 1A,1B: *PTPN11* (c.922A>G) triangular face and ptosis; Patient 2A,2B: *PTPN11* (c.184T>G), ptosis and low-set ears. Patient 3A: *PTPN11* (c.236A>G) down slanting palpebral fissures, low-set ears, ptosis; Patient 4A: *BRAF* (c.1861A>G) coarse facial features and down slanting palpebral fissures; Patient 5A: *MAPK21* (c.154G>C) low-set ears, hypertelorism; Patient 6A,6B,6C: RAF1 (c.786T>A) prominent forehead, ptosis, low-set ears and down slanting palpebral fissures along with melanotic macules on face and digit anomalies; Patient 7A: *SOS1* (c.1300G>A) coarse face, hypertelorism, low-set ear, ptosis; Patient 8A,8B: *SOS1* (c.1654A>G) coarse face, hypertelorism, low set ear, ptosis, pectus excavatum; Patient 9A: *NRAS* (c.34G>C) coarse face, short neck, broad nasal bridge; Patient 10A: *HRAS* (c.34G>C) triangular face, broad forehead, low set ears.

We recruited 84 patients with clinical suspicion of various syndromes in the spectrum of RASopathies (including NS, Noonan syndrome with multiple lentigines, CFCS, and Costello syndrome, NF1, etc.) between September 2021- September 2023 as per the flow chart (Figure 1). These patients were well clinically characterised, and representative pictures of a few of them (with informed consent taken) are given in Figure 2. Demographic details, relevant history, and complete physical examinations for major and minor anomalies were recorded by trained clinical geneticists. Ethical clearance was obtained from the ethics committee (Ref. No.: INT/IEC/2021/SPL-94). Informed consent for testing under research and the publication of data was obtained in accordance with the protocol. All genetic data were de-identified and handled, and participants had the right to withdraw consent at any time. All patients received pretest genetic counselling by certified clinical geneticists along with a detailed history and examination. Post-test counselling was provided after the results to interpret the findings, discuss any additional tests required, and explore management options. All patients had a normal standard karyotype. DNA from peripheral blood samples was extracted using QIAamp DNA Blood Midi Kit (Qiagen, Hilden, Germany) as per the manufacturer’s guidelines. Extracted DNA quality and quantity were checked using the Nanodrop instrument for further downstream processing.

### 2.2 Genetic testing

#### 2.2.1 Whole exome sequencing (WES)

WES was carried out using the Illumina HiSeq X10 platform (Illumina, Inc., San Diego, CA, United States). Here, DNA was sheared to produce 150–250 bp fragments for library preparation. It was used to perform targeted gene capture using a custom capture kit, following size selection, end-repair, phosphorylation, and adapter ligation to the DNA fragments according to the manufacturer’s protocol. Exome Library QC was checked on a Bioanalyzer (Agilent, United States) and quantified using Qubit (Invitrogen, United States). The libraries were sequenced as paired-end reads (2 × 150) for ∼80–100× coverage on HiSeq X (Illumina, CA).

### 2.3 Data analysis

Sequence reads were aligned to the human genome assembly GRCH38 using the Burrows–Wheeler Aligner (BWA) with the MEM algorithm (Li and Durbin, 2010). Variant calling and data processing were carried out using the Genome Analysis Toolkit (GATK, version 4.0,4.0 Broad Institute, https://software.broadinstitute.org/gatk/) McKenna. Variant annotation was performed using ANNOVAR software (http://annovar.openbioinformatics.org/en/latest/) (Yang and Wang, 2015). Filtering criteria used to narrow down the search of the causative variant: Synonymous, non-frameshift, or unknown variants, and low-quality reads were removed; frequency cut-off for population databases was set as <1% in dbSNP (http://www.ncbi.nlm.nih.gov/snp/), 1000 Genomes (http://browser.1000genomes. org/index.html), ESP6500 (http://evs.gs.washington.edu/EVS/), Exome Aggregation Consortium (ExAC; http://exac.broadinstitute.org/), and the Genome Aggregation Database (gnomAD; http://gnomad.broadinstitute. org/). Insilico analysis tools: PolyPhen2 (http://genetics.bwh. harvard.edu/pph2/), SIFT (http://sift.jcvi.org/), MutationTaster (http://mutationtaster.org/), and FATHMM-MKL (http://fathmm.biocompute.org.uk/fathmmMKL.htm) were used for variant effect prediction. ClinVar (ncbi.nlm.nih.gov/clinvar/), Human Genome Mutation Database: HGMD (https://www.hgmd.cf.ac.uk/ac/all.php), and DECIPHER database (https://decipher.sanger.ac.uk/) were used for identifying the reported or known SNVs and CNVs and the associated phenotypes. Variants identified were classified as per the latest ACMG guidelines (Richards et al., 2015; Tavtigian et al., 2018).

Categorical data were summarised as frequencies and percentages, and associations between genotype and phenotype were assessed using likelihood ratios with 95% confidence intervals. Statistical calculations were performed using IBM SPSS Statistics v26.0.

Additionally, exploratory drug–gene interaction mapping was performed using the Drug–Gene Interaction database (DGIdb v5.0.9).

## 3 Results

We enrolled all cases during the study period of 2 years, where RASopathy was suspected as the primary diagnosis. In total, we enrolled 84 cases. WES was done in all, but 31 of these turned out negative of a pathogenic or likely-pathogenic mutation. Of the 53 mutation-positive cases, 48 carried RASopathy-causing mutations, whereas the remaining 5 were consistent with having other genetic syndromes. Out of 48 cases with RASopathy-causing mutations, 14 were NF1 and have been described in a separate publication by the authors (Srivastava et al., 2024). Here we describe the 34 mutation-positive cases with RASopathy (excluding NF1), to put forward the molecular spectrum of RASopathies, as well as some other syndromes (n = 5) which may clinically mimic RASopathies owing to their overlapping features (Table 3).

### 3.1 Phenotype evaluation and demography

Of the thirty-four proven RASopathy cases, eighteen were male and sixteen were female. Three out of 34 (9%) were adults, and the remaining 31 (91%) were children. The mean age at diagnosis among adults was 29 years. Median age at diagnosis among children was 36 months (IQR 12–84 months) with a range of 0–192 months. Gender distribution was almost equal, with 47% females. The most common phenotypic features were short stature (64.7%), down slanting palpebral fissures (38.23%), chest wall deformity (35.29%), pulmonary stenosis (29.4%), and ptosis (29.4%). These signature phenotypic findings are commonly seen in cases of RASopathies disorders. Two patients had malignancies: JMML (Juvenile myelomonocytic leukaemia) and giant cell tumour (GCT) of knee.

### 3.2 Clinical features

**FIGURE 3:**
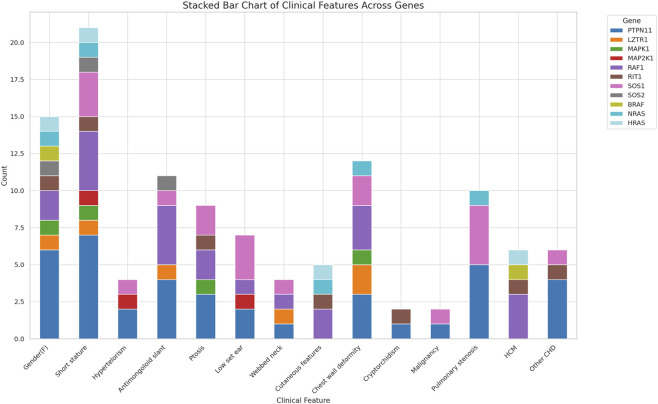
Summary of clinical features seen in patients with variants in different genes.

The common clinical features have been summarised in Table 1 and Figure 2. Some features of facial dysmorphism were present in almost all cases (Figure 2). No single clinical feature was found to be present consistently in all cases, showcasing its clinical heterogeneity. Indication of referral for adult cases was neuropsychiatric issues and a history of antenatal losses to foetal hydrops. A summary of clinical features seen in patients with variants in different genes is given in Figure 3.

**TABLE 1:** Phenotypic features and demography of mutation-positive cases.

Phenotype/Gene	*PTPN11* (n = 12)	*LZTR1* (n = 2)	*MAPK1* (n = 1)	*MAP2K1* (n = 1)	*RAF1* (n = 5)	*RIT1* (n = 2)	*SOS1* (n = 5)	*SOS2* (n = 1)	*BRAF* (n = 2)	*NRAS* (n = 1)	*HRAS* (n = 2)	Total (n = 34)
Gender (Female) (Male)	6	1	1	0	3	1	0	1	1	1	1	16 (47%)
	6	1	1	1	2	1	5	0	1	0	1	18 (53%)
Short stature	8	1	1	1	4	1	3	1	0	1	1	22 (64.7%)
Down slanting palpebral fissures	5	1	0	0	5	0	1	1	0	0	0	13 (38.23%)
Chest wall deformity	3	2	1	0	3	0	2	0	0	1	0	12 (35.29%)
Pulmonary stenosis	5	0	0	0	0	0	4	0		1	0	10 (29.4%)
Ptosis	4	0	1	0	2	1	2	0	0	0	0	10 (29.4%)
Low-set ear	3	0	0	1	1	0	3	0	0	0	0	8 (23.5%)
HCM	0	0	0	0	4	1	0	0	1	0	1	7 (20.59%)
Other CHD (ASD/VSD/PDA/TOF)	4	0	0	0	1	1	1	0	0	0	0	7 (20.59%)
Webbed neck	2	1	0	0	1	0	1	0	0	0	0	5 (14.7%)
Cutaneous features	0	0	0	0	2	1	0	0	0	1	1	5 (14.7%)
Hypertelorism	2	0	0	1	0	0	1	0	0	0	0	4 (11.76%)
Cryptorchidism	1	0	0	0	0	1	0	0	0	0	0	2 (5.9%)
Malignancy	1 (JMML)	0	0	0	0	0	1 (GCT)	0	0	0	0	2 (5.9%)

CHD (congenital heart defect), HCM (Hypertrophic Cardiomyopathy), ASD (Atrial Septal Defect), VSD (Ventricular Septal Defect), PDA (Patent Ductus Arteriosus), TOF (Tetralogy of Fallot), JMML (Juvenile myelomonocytic leukaemia), GCT (giant cell tumour).

### 3.3 Mutation analysis

**FIGURE 4:**
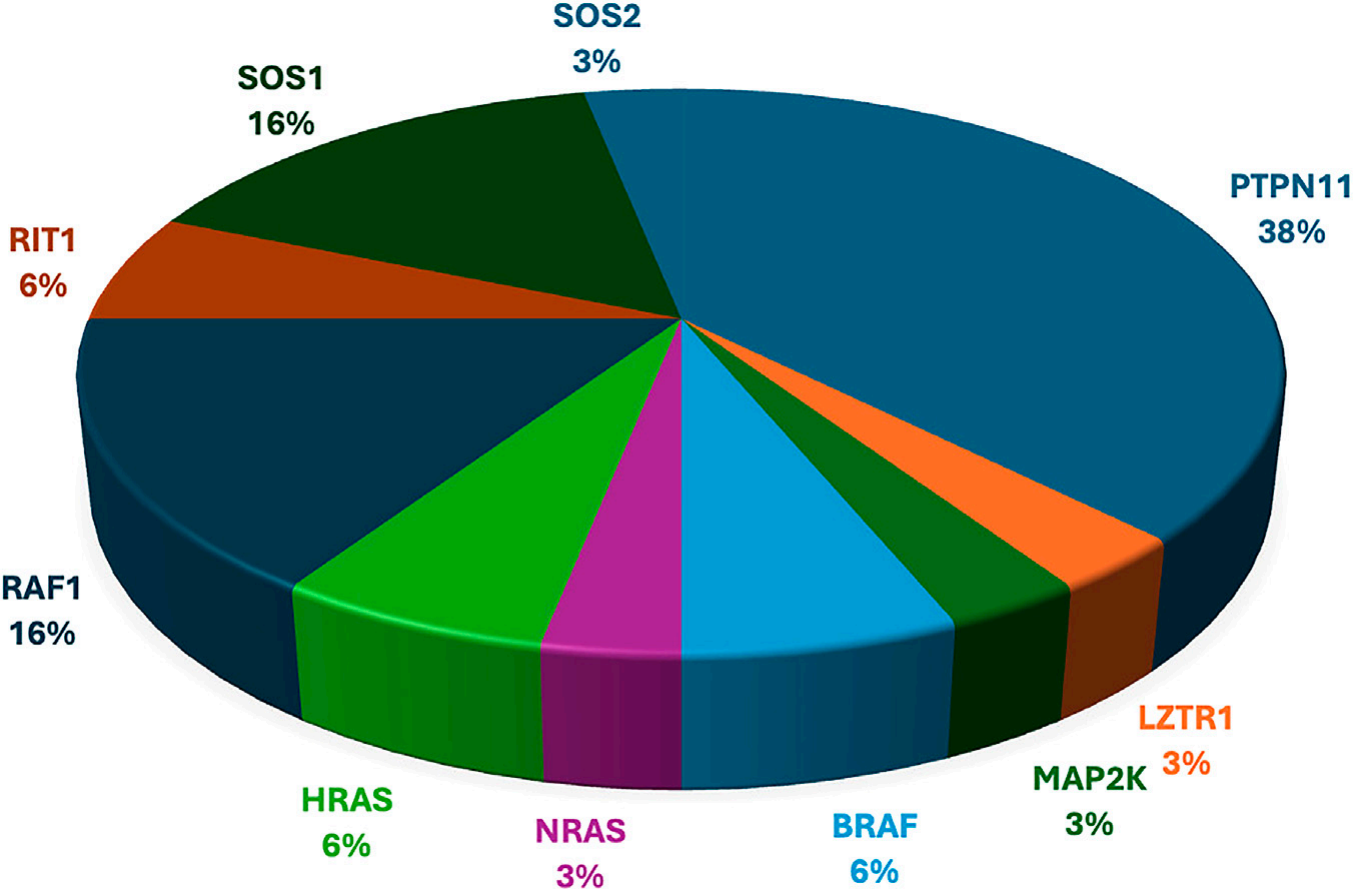
Distribution of variants in various RASopathy genes identified in our cohort.

**FIGURE 5:**
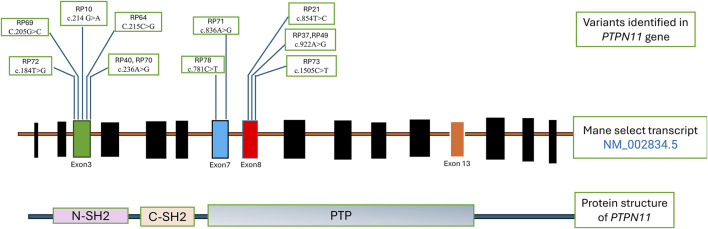
Distribution of variants in *PTPN11* gene.

Table 2 shows the cases where pathogenic/likely pathogenic variants in the RAS/MAPK pathway genes were identified. Two variants of uncertain significance (VUS) are presented separately in Supplementary Table S1. The *NF1* gene, although a part of the pathway, leads to a clinically distinct phenotype and hence has not been included in this study. The spectrum of cases of neurofibromatosis from our group has been published previously (Srivastava et al., 2024). All variants were missense and heterozygous, thus inherited in an autosomal dominant fashion. Genes in which variants were identified were *PTPN11, LZTR1, MAPK1, MAP2K, RAF1, RIT1, SOS1, SOS2, BRAF, NRAS, HRAS*. More than 38% cases (12 cases) had variants in *PTPN11*, 5 had variants in *SOS1* and *RAF1* each, making these three the most common genes leading to RASopathy disorders (Figure 4). *PTPN11* gene variants were most common owing to being readily identifiable phenotypic findings, such as short stature (66.67%), down slanting palpebral fissures (41.67%), and pulmonary stenosis (41.67%) in many patients. Exons 3,7,8 were most commonly involved with maximum cases in exon 3, indicating it could be a hotspot in the North Indian population. The position of the variants identified in the *PTPN11* gene in our study is represented in Figure 5. Hotspots for other genes could also be identified for, e.g.,: *HRAS* (exon 2), *RAF1* (exon 7), *SOS1* (exon 10). Details of all variants, protein change, ACMG criteria met, ACMG ClinGen scoring of each variant, and final classification are given in Table 2 and Supplementary Table S1. Three variants were novel, and the rest of the variants were already known variants with Clingen-specific guidelines.

**TABLE 2:** Details of pathogenic/likely pathogenic variants identified.

Patient ID	Gene (Transcript id)	c. Number	Exon	Type of variant	Amino acid change	ACMG-Clingen scoring	Pathogenicity score as per Clingen-ACMG guidelines	Final classification	Clinvar ID/Novel
RP 72	*PTPN11* (NM_002834.5)	c.184T>G	3	Missense	p.Tyr62Asp	PP5(VS)/PM1(S)/PM5(S)/PP3(S)/PM2(Supporting)	21points = 21P-0B	Pathogenic	RCV000033466.27
RP69	*PTPN11* (NM_002834.5)	c.205G>C	3	Missense	p.Glu69Gln	PP5(VS)/PM1(S)/PM5(S)/PP3(M)/PM2(Supporting)	19points = 19P-0B	Pathogenic	RCV000033469
RP10	*PTPN11* (NM_002834.5)	c.214 G>A	3	Missense	p.Ala72Thr	PP5(VS)/PM1(S)/PM5(S)/PP3(M)/PM2(Supporting)	19points = 19P-0B	Likely Pathogenic	VCV000177754.18
RP64	*PTPN11* (NM_002834.5)	c.215C>G	3	Missense	p.Ala72Gly	PP5(VS)/PM1(S)/PM5(S)/PP3(S)/PM2(Supporting)	21points = 21P-0B	Pathogenic	VCV000013325.51
RP40	*PTPN11* (NM_002834.5)	c.236A>G	3	Missense	p.Gln79Arg	PP5(VS)/PM5(S)/PP3(S)/PM1(S)/PM2 (Supporting)	21points = 21P-0B	Pathogenic	VCV000013340.43
RP 70	*PTPN11* (NM_002834.5)	c.236A>G	3	Missense	p.Gln79Arg	PP5(VS)/PM5(S)/PP3(S)/PM1(S)/PM2 (Supporting)	21points = 21P-0B	Pathogenic	RCV000014268.22
RP 78	*PTPN11* (NM_002834.5)	c.781C>T	7	Missense	p.Leu261Phe	PP5(VS)/PM1(M)/PM5(S)/PM2(Supporting)	15points = 15P-0B	Pathogenic	RCV000522926
RP 71	*PTPN11* (NM_002834.5)	c.836A>G	7	Missense	p.Tyr279Cys	PP5(VS)/PP3(S)/PM1(M)/PM5(M)/PS3(Supporting)/PM2(Supporting)	18points = 18P-0B	Pathogenic	RCV000077859.52
RP21	*PTPN11* (NM_002834.5)	c.854T>C	8	Missense	p.Phe285Ser	PP5(VS)/PM5 (5)/PP3(S)/PM1(M)/PM2 (Supporting)	19points = 19P-0B	Pathogenic	VCV000013335.41
RP 37	*PTPN11* (NM_002834.5)	c.922A>G	8	Missense	p.Asn308Asp	PP5(VS)/PM5(S)/PP3(S)/PM1(Mod)/PM2 (Supporting)	19points = 19P-0B	Pathogenic	VCV000013326.101
RP49	*PTPN11* (NM_002834.5)	c.922A>G	8	Missense	p.Asn308Asp	PP5(VS)/PM5(S)/PP3(S)/PM1(Mod)/PM2 (Supporting)	19points = 19P-0B	Pathogenic	VCV000013326.101
RP 73	*PTPN11* (NM_002834.5)	c.1505C>T	8	Missense	p.Ser502Leu	PP5(VS)/PM1(S)/PM5(S)/PP3(S)//PM2 (Supporting)	21points = 21P-0B	Pathogenic	RCV000033544.29
RP 74	*LZTR1* (NM_006767.4)	c.742G>A	8	Missense	p.Gly248Arg	PS3(VS)/PS1(S)/PM5(S)/PM1(M)/PP3(M)/PM2 (Supporting)/PP5(Supporting)	22points = 22P-0B	Pathogenic	RCV000191027.23
RP77	*MAP2K1* (NM_002755.4)	c.154G>C	2	Missense	p.Ala52Pro	PS2(S)/PM1(S)/PM2 (Supporting)	9points = 9P-0B	Likely Pathogenic	Novel variant (SUB15708038)
RP12	*BRAF* (NM_004333.6)	c.1406 G>A	11	Missense	p.Gly469Glu	PP5(VS)/PM1(S)/PM5(S)/PP3(S)/PM2(Supporting)	21points = 21P-0B	Pathogenic	VCV000013974.39
RP 75	*BRAF* (NM_001374258.1)	c.1861A>G	15	Missense	p.Asn581Asp	PP5(VS)/PM5(S)/PP3(S)/PM1(M)/PM2(Supporting)	19points = 19P-0B	Pathogenic	RCV000211751.7
RP27	*NRAS* (NM_002524.5)	c.34G>C	2	Missense	p.Gly12Arg	PM1(S)/PM5(S)/PP5(S)/PP3(M)/PM2(Supporting)	15points = 15P-0B	Pathogenic	VCV000040469.10
RP 76	*HRAS* (NM_005343.4)	c.34G>A	2	Missense	p.Gly12Ser	PP5(VS)/PM5(S)/PP3(S)/PM1(S)/PM2(Supporting)	21points = 21P-0B	Pathogenic	RCV000013435.78
RP79	*HRAS* (NM_005343.4)	c.37G>T	2	Missense	p.Gly13Cys	PP5(VS)/PM5(S)/PP3(S)/PM1(S)/PM2(Supporting)	21points = 21P-0B	Pathogenic	RCV000678903.4
RP61	*RAF1* (NM_002880.4)	c.770C>T	7	Missense	p.Ser257Leu	PS3(VS)/PM1(S)/PM5(S)/PM2(Supporting)/PP3(Supporting)	18points = 18P-0B	Pathogenic	VCV000013957.83
RP 80	*RAF1* (NM_002880.4)	c.770C>T	7	Missense	p.Ser257Leu	PS3(VS)/PM1(S)/PM5(S)/PM2(Supporting)/PP3(Supporting)	18points = 18P -0B	Pathogenic	RCV000157426.14
RP 81	*RAF1* (NM_002880.4)	c.770C>T	7	Missense	p.Ser257Leu	PS3(VS)/PM1(S)/PM5(S)/PM2(Supporting)/PP3(Supporting)	18points = 18P -0B	Pathogenic	RCV000157426.14
RP 68	*RAF1* (NM_001354689.3)	c.781C>G	7	Missense	p.Pro261Ala	PP5(VS)/PS3(S)/PM1(S)/PM5(S)/PP3(M)/PM2(Supporting)	23points = 23P-0B	Pathogenic	VCV000183408.23
RP53	*RAF1* (NM_002880.4)	c.786T>A	7	Missense	p.Asn262Lys	PS1(S)/PM1(S)/PM5(S)/PP5(S)/PM2(Supporting)	17points = 17P-0B	Pathogenic	VCV000044634.11
RP 82	*RIT1* (NM_006912.6)	c.244T>G	5	Missense	p.Phe82Val	PP5(VS)/PM1(S)/PM5(S)/PP3(S)/PM2(Supporting)/BP6(Supporting)	20points = 21P-1B	Pathogenic	RCV000170492.21
RP62	*RIT1* (NM_006912.6)	c.247A>C	5	Missense	p.Thr83Pro	PP5(VS)/PM1(S)/PP3(M)/PM2(Supporting)/BS3(Supporting)	14points = 15P-1B	Pathogenic	VCV000183409.17
RP83	*SOS1* (NM_005633.4)	c.1300G>A	10	Missense	p.Gly434Arg	PP5(VS)/PS1(S)/PM1(S)/PP3(S)/PM5(M)PM2(Supporting)	23points = 23P-0B	Pathogenic	RCV001270835.7
RP41	*SOS1* (NM_005633.4)	c.1654A>G	10	Missense	p.Arg552Gly	PP5(VS)/PM1(S)/PM5(S)/PP3(S)/PM2(Supporting)	21points = 21P-0B	Pathogenic	VCV000012871.54
RP75	*SOS1* (NM_005633.4)	c.1654A>G	10	Missense	p.Arg552Gly	PP5(VS)/PM1(S)/PM5(S)/PP3(S)/PM2(Supporting)	21points = 21P-0B	Pathogenic	RCV000156980
RP-13	*SOS1* (NM_005633.4)	c.1655 G>A	10	Missense	p.Arg 552Lys	PP5(VS)/PM1(S)/PM5(S)/PP3(M)/PM2(Supporting)	19points = 19P-0B	Pathogenic	VCV000040683.34
RP-84	*SOS1* (NM_005633.4)	c.2104T>C	13	Missense	p.Tyr702His	PP5(S)/PM5(M)/PP3(M)/PM2(Supporting)	9points = 9P-0B	Likely pathogenic	RCV000159124
RP48	*SOS2* (NM_006939.4)	c.800T>G	6	Missense	p.Met267Arg	PM5(S)/PP5(S)/PM1(M)/PP3(M)/PM2(Supporting)	13points = 13P-0B	Pathogenic	VCV000577079.18

All the variants were heterozygous for autosomal dominant conditions.

### 3.4 Novel variants

The missense variant NM_002745.5 (*MAPK1*):c.128A>T (p.Tyr43Phe) has not been reported previously as a pathogenic variant nor as a benign variant, to our knowledge. The p.Tyr43Phe variant is novel (not in any individuals) in gnomAD and 1 kG. There is a small physicochemical difference between tyrosine and phenylalanine. The gene *MAPK1* has a low rate of benign missense variation as indicated by a high missense variants Z-Score of 3.61. Missense Variants Z-Score is produced by the Exome Aggregation Consortium (60,706 adult humans) by computing a signed Z score for the deviation of observed counts from the expected number. For these reasons, this variant has been classified as uncertain significance. This variant has been submitted in ClinVar (SUB15708036) (Supplementary Table S1).

Another missense variant NM_006767.4 (*LZTR1*):c.824G>A (p.Arg275Gln) has not been reported previously as a pathogenic variant nor as a benign variant, to our knowledge. The p.Arg275Gln variant is novel (not in any individuals) in 1 kG. There is a small physicochemical difference between arginine and glutamine, which is not likely to impact secondary protein structure as these residues share similar properties. The p.Arg275Gln missense variant is predicted to be damaging by both SIFT and PolyPhen2. For these reasons, this variant has been classified as uncertain significance. This variant has been submitted in ClinVar (SUB15708030) (Supplementary Table S1).

*MAP2K1* (p.Ala52Pro) variant is not reported in gnomAD/1 kG. Non-truncating non-synonymous variant is located in a mutational hot spot and/or critical and well-established functional domain. missense mutation is a common mechanism of this disease. Computational prediction tools unanimously support a deleterious effect on the gene. As per ACMG guidelines, the scoring indicates a likely pathogenic (Table 2). This variant has been submitted in ClinVar (SUB15708038). Although segregation analysis could not be performed for these variants due to the unavailability of parental samples.

### 3.5 Genotype-phenotype correlation

Most *PTPN11*-positive cases showed short stature and facial dysmorphism (prominent eyes, downslanting palpebral fissures, coarse facies). One patient (RP72; c.184T>G) had tetralogy of Fallot, suggesting this rare variant may confer a more severe cardiac phenotype. ASD and VSD occurred in three cases—RP40 (c.236A>G), RP70 (c.236A>G), and RP73 (c.1505C>T)—findings atypical for *PTPN11*-associated Noonan syndrome. RP71 (c.836A>G) presented with early-onset hypertrophic cardiomyopathy. A neonate (RP78; c.781C>T) was diagnosed antenatally via trio exome sequencing, confirming paternal inheritance of the variant; given the father’s mild phenotype, pregnancy was continued. One case (RP70) had JMML with recurrent c.236A>G mutation (COSMIC ID: COSV61004775). Another infant (RP21; c.854T>C) had antenatal cystic hygroma and was later enrolled.

A 32-year-old female (RP74; *LZTR1* c.742G>A) presented with pituitary macroadenoma (12 × 6 × 7 mm, internal carotid artery (ICA) invasion)—an uncommon feature in *LZTR1*-related Noonan syndrome—alongside facial dysmorphism but normal stature, intellect, and cardiac function. These findings were unique in relation to *LZTR1*. RP75 (*BRAF* c.1861A>G) showed cardio-facio-cutaneous features with normal echocardiography, an atypical finding. RP27 (*NRAS* c.34G>A) exhibited prenatal polyhydramnios, narrow thorax, pulmonary hypoplasia, and postnatal myxomatous cardiac valves, rarely reported in Noonan syndrome 6. Among five *RAF1* cases, four had exon 7 mutations and adolescent-onset cardiac symptoms, suggesting a cardiac-predominant hotspot. Four of five *SOS1* cases had exon 10 variants, with consistent short stature, ptosis, and pulmonary stenosis, reaffirming known genotype–phenotype correlations.

No significant correlation was noted between phenotype and the gene of origin, except in the cases of pulmonary stenosis associated with *PTPN11* and *SOS1*. A positive likelihood ratio of 3.67 (95% CI: 0.93-15) and a negative likelihood ratio of 0.41 (95% CI: 0.13-1.31) was calculated which was not significant.

### 3.6 Other syndromes identified with overlapping phenotypes

Since the enrolment for this study was clinically directed and not based on the molecular report, we were able to identify cases that have overlapping features with RASopathies. Out of the enrolled cases, five came out to be positive for a syndrome which was not a RASopathy (Table 3). However, no two such cases shared the same gene, signifying that these are either common diagnoses rarely mimicking RASopathies or rare diagnoses by themselves. In the diagnoses, CHARGE and Stickler Syndrome were identified in two cases (RP-2 and RP-36). They could be considered as two relatively common diagnoses in the population, with an incidence of around 1 in 10000. The remaining three individuals were diagnosed with Zellweger spectrum disorder (RP-18), Koolen-De Vries syndrome (RP-45), and Coffin-Lowry syndrome (RP-39), respectively. Comparative phenotypic analysis revealed that downslanting palpebral fissures were the most common overlapping feature (present in 4 of the 5 cases), followed by cardiac defects (n = 4), short stature (n = 3), and pectus anomalies (n = 2).

**TABLE 3:** Table showing variants in genes other than RASopathies spectrum in mimic patients.

Patient ID	Age/Gender (M = Male, F=Female)	Presenting complaint	Pointers leading to clinical misdiagnosis	Gene	Variant details	ACMG clingen scoring	Pathogenicity score as per Clingen-ACMG guidelines	Final variant classification	Zygosity/mode of inheritance	OMIM phenotype/Mode of inheritence
RP-2	2 Month/M	Microcephaly, small length for age, facial dysmorphism in the form of downslanting palpebral fissures, Ventricular septal defect	Down slanting palpebral fissures	*CHD7* (NM_017780.4)	c.469C>T (p.Arg157Ter)	PVS1(VS), PP5(VS), PM2 (Supporting)	17points = 17P-0B	Pathogenic	Heterozygous AD	CHARGE syndrome (# 214800)
RP-18	1 year/M	Global developmental delay, hypotonia, microcephaly, failure to thrive, cafe au lait spots, left undescended testis subtle dysmorphism- forehead balding, down slanting palpebral fissures and low set ears	Down slanting palpebral fissures, low set ears	*PEX2* *(*NM_000318.3)	c.665C>A (p.Ser222Ter)	PVS1(VS), PM2(Supporting)	9points = 9P-0B	Likely Pathogenic	Compound Heterozygous AR	Peroxisome biogenesis disorder 5A (Zellweger)/Peroxisome biogenesis disorder 5B (614866/614867) AR
					c.570C>A (p.Tyr190Ter)	PVS1(S), PM2 (Supporting) PP5(Supporting)	6points = 6P-0B	Likely Pathogenic		
RP-36	5 years/F	SGA at birth, facial dysmorphism, short stature, downslanting palpebral fissures, pectus excavatum, atrial septal defect	Short stature, down slanting palpebral fissures, pectus excavatum	*COL2A1* (NM_001844.5)	c.3627_3644del (p.Pro1211_1216del)	PS2(S) PM2 (Supporting), PM4(M) PM1(Supporting)	8points = 8P-0B	Likely Pathogenic	Heterozygous AD	Stickler syndrome type 1 (# 108300)
RP-39	7 years/M	GDD, microcephaly, short stature, downslanting palpebral fissures, primary pulmonary arterial Hypertension	Short stature, down slanting palpebral fissures	*RPS6KA3* *NM_004586.3*	c.1777C>T (p.Gln593Ter)	PVS1(VS), PM2 (Supporting)	9points = 9P-0B	Likely Pathogenic	Hemizygous XLD	Coffin-Lowry syndrome XLD (# 303600)
RP-45	1 year/F	Presented with facial dysmorphism, frontal bossing, curly hair, pointed ears, pectus excavatum, autism spectrum disorder	Pectus excavatum, curly hair	*KANSL1* *NM_015443.4*	c.1760_1770del (p.Thr587Serfs*10)	PVS1(very strong), PM2 (Supporting)	9points = 9P-0B	Likely Pathogenic	Heterozygous AD	Koolen-De vries syndrome #610443

AD: autosomal dominant, AR: autosomal recessive.

### 3.7 DGIdb analysis

The results of the gene–drug interaction analysis, summarised in Supplementary Table S2 and Supplementary Figure S1, highlight several significant interactions between clinically relevant genes and FDA-approved therapeutic agents. Among the interactions, genes like *RIT1* and *NRAS* demonstrated notably high interaction scores with targeted inhibitors such as Selumetinib and Dabrafenib.

## 4 Discussion

This study presents a comprehensive clinico-genetic analysis of a cohort of 84 North Indian patients with clinical suspicion of RASopathy spectrum disorders, identifying causative germline activating variants in 53 cases. Our findings emphasize the clinical and genetic heterogeneity of RASopathies, reaffirm the central role of the RAS/MAPK signalling pathway in their pathogenesis, and offer insights into potential therapeutic avenues through drug–gene interaction analysis.

Among the confirmed RASopathy cases, the balanced gender distribution and predominant representation in early childhood (mean age, 3 years) align with the age of onset and diagnosis patterns reported in the global literature (Athota et al., 2020; Yu et al., 2019). Short stature (64.7%), cardiac anomalies (70%), and facial dysmorphism (almost all cases had some form of dysmorphism) were the most prevalent clinical features. This profile mirrors the findings of Yu et al. (2019), who described short stature (65.9%) and cardiac involvement (65.7%) as dominant traits in a large Chinese cohort (Yu et al., 2019). Cardiac findings in our study were predominantly pulmonary stenosis and hypertrophic cardiomyopathy—patterns that again align well with prior reports (Tartaglia et al., 2001). The clinical heterogeneity observed in our cohort, with no single feature universally present, reaffirms the diagnostic complexity associated with these syndromes. Intellectual disability was seen in 29.4% of our cases, slightly lower than Yu et al.’s 36.9%, possibly reflecting regional variation or ascertainment differences. Among facial features, downslanting palpebral fissures emerged as the most consistent dysmorphism, present in nearly half the cases. Skeletal anomalies, such as pectus deformity and short neck, were common, while skin manifestations, including café au lait spots and haemangiomas, were noted in a subset, indicating partial phenotypic overlap with disorders such as NF1. The occurrence of JMML and a giant cell tumour in two cases highlights the known oncogenic predisposition associated with some RASopathies (Kratz et al., 2015).

The observed diagnostic yield of ∼63% (53/84) aligns with previously reported yields ranging between 40% and 70% using Whole Exome Sequencing (WES) in similarly suspected cohorts (Rauen, 2013; Kouz et al., 2016). The predominance of *PTPN11* mutations (38% of positive cases) is consistent with its known association with Noonan syndrome and its frequent implication in RASopathies globally (Tartaglia et al., 2001; Roberts et al., 2013). In comparison to global trends, our cohort showed a notably high proportion of Noonan syndrome (NS) cases attributed to pathogenic variants in *SOS1* and *RAF1*, with each accounting for 16% of cases. This elevated frequency suggests the possibility of population-specific or founder variants in the Indian population contributing to NS (Roberts, 2001). In our cohort, missense mutations were the exclusive variant type detected, underscoring the activating nature of pathogenic alterations in RASopathy genes. This finding also reinforces the gain-of-function mechanism underlying the majority of these disorders, which differ from tumour suppressor–related genetic conditions where loss-of-function variants predominate (Tidyman and Katherine, 2016).

The observed trend linking *PTPN11* mutations to pulmonary stenosis, although statistically inconclusive due to sample size constraints, is biologically plausible and has been echoed in earlier genotype–phenotype studies (Zenker et al., 2004). *SOS1* was also significantly associated with pulmonary stenosis in our cohort, which has been reported in Korean patients in the past (Lee et al., 2011). Other reported associations, such as hypertrophic cardiomyopathy with *RAF1* mutations and ectodermal anomalies with *HRAS* mutations (Costello syndrome), were also partially recapitulated in our cohort, further validating established phenotypic correlations (Pandit et al., 2007; Kerr et al., 2006).

The usual age of diagnosis of RASopathy disorders is childhood. In general, patients get diagnosed in the first decade of life due to growth failure, cardiac findings, cutaneous features and dysmorphism, which evolve over time (Digilio et al., 2006; 2011). Diagnosis in adulthood can be challenging, but a high index of suspicion leads to a correct approach. Among the three adults, two were referred due to neuropsychiatric issues not responding to conventional treatment (RP 69, RP41). Characteristic facial gestalt, short stature and pectus deformity helped to reach a diagnosis of NS. Neuropsychiatric issues have been known to be present in more than half of cases in NS (Naylor). The third case was a female who came for pre-conceptional counselling with a history of two early pregnancy losses due to non-immune hydrops (RP74). Upon evaluation, she was found to have a webbed neck, pectus deformity and a history of pituitary microadenoma resection in past. Genetic testing revealed a variant in *LZTR1*, leading to the diagnosis of NS 10. Moreover, *LZTR1* variant has been previously reported to be associated with severe developmental delay and intellectual disability (Pagnamenta et al., 2019; Chinton et al., 2019), both of which were absent in this case, supporting a broader phenotypic spectrum or variable expressivity. Hence novel phenotypic findings like normal intellect and pituitary macroadenoma would expand phenotypic spectrum associated with *LZTR1* variant. Hence it can be challenging to diagnose this group of patients when presenting in adulthood (Zenker et al., 2022).

Reverse phenotyping revealed several atypical genotype–phenotype correlations. A patient (RP75; *BRAF* c.1861A>G) displayed classical cardiofaciocutaneous features but had normal cardiac findings—unusual for *BRAF*-related CFCS. Another patient (*NRAS* c.34G>A), typically associated with somatic cancers (Abdelgadir et al., 2025), showed prenatal polyhydramnios, narrow thorax, pulmonary hypoplasia, and postnatal myxomatous cardiac valves, expanding the *NRAS* germline phenotype (Altmüller et al., 2017). Among two *HRAS* hotspot cases (p.Gly12Ser, p.Gly13Cys) consistent with Costello syndrome, one presented with severe short stature, brachydactyly, and normal cardiac profile, initially mimicking hypochondroplasia—underscoring the importance of detailed phenotypic evaluation in mild skeletal dysplasia–like presentations. Four of five *RAF1*-mutant patients developed adolescent-onset cardiac manifestations (dyspnea, palpitations), supporting *RAF1*-related Noonan syndrome as a differential for hypertrophic obstructive cardiomyopathy. Similarly, four of five *SOS1* cases harbored exon 10 variants with consistent short stature, ptosis, and pulmonary stenosis, reaffirming established associations (Pandit et al., 2007). Overall, these findings illustrate both expected and novel phenotypic spectra across RASopathy-related variants, emphasising the value of integrated genotype–phenotype correlation for accurate diagnosis, counselling, and clinical management.

Prenatal diagnosis of RASopathies has been reported in very few cases in the literature. The main reason is non-specific phenotypic findings and poor detection rates of these findings, especially in resource-limited settings. Increased nuchal translucency and polyhydramnios have been the indicators leading to suspicion of Noonan syndrome in foetuses. Using NGS as a modality of testing in antenatal cases is not yet widespread in clinical practice. Hence, even in cases with these markers, chromosomal anomalies and common infections like TORCH are traditionally ruled out. One of the cases (RP68) in our cohort had a history of increased NT in the first trimester. Amniocentesis followed by Trio exome (foetus and both partners) was offered to the family. The foetus, as well as the male partner, was found to be carrying a pathogenic variant in *PTPN11*. Reverse phenotyping revealed multiple affected family members on the paternal side. After appropriate genetic counselling, the family opted to continue the pregnancy as the variant was associated with a milder phenotype and the quality of life expectancy within the family was not affected (Pannone et al., 2017). Hence, genetic testing could help in informed decision-making for the family, and the family was put on surveillance.

The mutational landscape in our cohort was consistent with previously reported data, with *PTPN11* being the most commonly affected gene. Twelve patients had *PTPN11*-related Noonan Syndrome, and exon 3 emerged as a recurrent hotspot. While exon 13 mutations were reported in prior Indian studies (Athota et al., 2020). Our cohort uniquely featured multiple exon 7, exon 8 variants, aligning more closely with East Asian data (Yu et al., 2019). The recurrence of the c.922A>G variant in two patients further supports it as a likely common pathogenic allele in the Indian population. The absence of nonsense or frameshift variants supports the hypothesis that *PTPN11* mutations are generally missense and act via gain-of-function mechanisms, with the gene being intolerant to haploinsufficiency (Rauen, 2013; Athota et al., 2020). In addition, variants were identified in a wide range of other RASopathy-associated genes, including *LZTR1, BRAF, SOS1*, and *RAF1*, underscoring the necessity of broad genetic testing approaches such as WES in cases with overlapping phenotypes.

A significant insight from this study is the frequency with which non-RASopathy disorders clinically mimicked RASopathies. Of the cases recruited based on clinical suspicion, five were ultimately found to have other syndromes, including CHARGE and Stickler syndrome (Table 3). Interestingly, downslanting palpebral fissures, short stature, curly, sparse hair, and chest wall anomalies were common, misleading features. Skin and hair involvement, significant neurodevelopmental impairment, and the presence of microcephaly were also more prevalent in the non-RASopathy cohort. Ptosis and pulmonary stenosis have previously been documented to be significantly associated with RASopathies spectrum (Bhoj et al., 2017). None of them had ptosis, pulmonary stenosis, digital anomalies, tumour/cancers, or short neck when compared to RASopathy cases. Short stature and facial dysmorphism were seen in more than 2/3rd cases, which usually leads to clinical misdiagnosis (Lallar et al., 2021). Hence, clinical labelling of RASopathy spectrum based on these subtle common findings alone should be avoided, and more specific findings like ptosis, pulmonary stenosis, and HCM should be actively looked for.

The findings reinforce the importance of using NGS, especially in patients with overlapping or ambiguous phenotypes. Our ability to detect pathogenic variants in genes such as *LZTR1, MAPK1*, and *SOS1* supports the utility of WES in uncovering less common causes of RASopathies. Moreover, early molecular diagnosis can facilitate surveillance for associated complications and emerging genotype-specific therapies, such as MEK inhibitors (Gross et al., 2020; Dombi et al., 2016).

Among the enrolled patients, all exhibited clinical features consistent with the RASopathy spectrum, but 31 patients yielded negative results on WES. The most common phenotypic features prompting inclusion were short stature, facial dysmorphism, ectodermal abnormalities (e.g., curly or sparse hair), cutaneous findings such as café-au-lait macules (CALMs), pectus deformities, congenital heart defects, malignancies, and developmental delays. Notably, many of these negative cases had strong clinical suspicion for RASopathy, and in several instances, the phenotype was re-evaluated to confirm alignment with the spectrum. The absence of a molecular diagnosis in these cases may be attributed to several limitations of WES, including the inability to capture deep intronic variants, regions with poor coverage, copy number variants (CNVs), epigenetic alterations, or mutations in regulatory regions, as well as mosaicism that falls below the detection threshold of WES. These findings highlight the need for comprehensive reanalysis, potential use of complementary genomic technologies (e.g., whole genome sequencing, CNV analysis), and deeper phenotyping in cases with strong clinical indicators but negative genetic results.

As a secondary exploratory component, we queried the Drug–Gene Interaction database (DGIdb) to assess whether the genes identified in our cohort have known or predicted interactions with existing drugs. This analysis revealed multiple candidate drug–gene associations, including MEK inhibitors and SHP2 modulators, consistent with prior literature on targeted therapy development in RASopathies. The detailed list of interactions (Supplementary Table S2) and a schematic network (Supplementary Figure S1) are presented as hypothesis-generating findings. While these results may inform future research directions, they are not intended as clinical recommendations, given the lack of functional validation or therapeutic testing in our study.

Despite these promising leads, challenges remain. Most RASopathies manifest in early development, and long-term safety data for chronic use of kinase inhibitors in paediatric populations are lacking. Moreover, the pleiotropic effects of RAS pathway modulation necessitate careful patient selection and surveillance (Romano et al., 2010). Clinical trials specifically targeting non-NF1 RASopathies remain limited, and future studies must address dosage, timing, and efficacy endpoints in these rare conditions. From a diagnostic standpoint, our study reinforces the value of WES as a frontline tool in evaluating patients with syndromic features and suspected RASopathy. Particularly in resource-limited settings, sequencing strategies that encompass a broad spectrum of candidate genes may prove more efficient than phenotype-driven single-gene testing. This is especially pertinent for cases with overlapping clinical signs or atypical presentations, which constituted a significant subset in our cohort. Genetic counselling is critical in the context of rare disease diagnosis, particularly when genomic testing is involved. Our framework ensured that patients and families were adequately informed and supported throughout the diagnostic journey, aligning with best-practice guidelines and mitigating the psychosocial impact of uncertain or incidental findings. Another strength of our approach is the focus on the North Indian population, where data on RASopathies remains sparse. Our findings contribute to the global diversity of reported pathogenic variants and highlight potential population-specific patterns. For instance, recurrent mutations in *PTPN11* (e.g., p.Asn308Asp, p.Gln79Arg) observed in our cohort are also among the most common worldwide, while novel or rare variants in *LZTR1* and *RIT1* may merit further functional validation in population-specific contexts.

However, this study has certain limitations. The cohort size was modest; larger cohorts are needed to enable more robust genotype–phenotype associations. Long-term clinical follow-up data were not available for all patients. Functional validation of novel variants and assessment of drug efficacy in clinical trials or patient-derived cell models would further strengthen our findings. Moreover, the bioinformatics approach identifies potential targets but requires pharmacogenomic validation and careful clinical assessment.

## 5 Conclusion

In conclusion, our study provides important insights into the clinico-genetic landscape of RASopathies in Indian patients and emphasizes the utility of WES in diagnosing syndromes with significant phenotypic overlap. Our exploratory use of drug–gene interaction databases identified potential candidate interactions with FDA-approved agents, suggesting possible avenues for therapeutic repurposing. However, these findings should be regarded as hypothesis-generating and not as clinical guidance, given the absence of functional validation or therapeutic testing in our cohort. Future research integrating molecular, functional, and clinical studies will be essential to establish the true therapeutic potential of these interactions. Disclaimer: The drug–gene interaction findings are exploratory and hypothesis-generating; no therapeutic testing was performed in this study.

## Data Availability

The datasets presented in this study can be found in online repositories. Data associated with this study are available in a publicly available repository, ClinVar (https://www.ncbi.nlm.nih.gov/clinvar/), and accession numbers are given in Table 2.

## Supplementary material

SUPPLEMENTARY FIGURE S1 Exploratory DGIdb-derived drug–gene interaction network.
