# Supplementary material for: Germline activating sequence variations in RASopathy spectrum genes: genotype–phenotype correlation in a North Indian cohort
Source: Front Genet. 2025 Nov 11;16:1677143. doi: 10.3389/fgene.2025.1677143 (PMC12643386; doi:10.3389/fgene.2025.1677143)
Supplement: Supplementary file 1 [file Table1.docx]

**Supplementary Table 1:** Table showing variants of unknown significance (VUS) in RASopathy genes

| **Patient ID** | **Gene (Transcript id)** | **c. number** | **Exon** | **Type of variant** | **Amino acid change** | **ACMG-Clingen scoring** | **Pathogenicity Score as per Clingen-ACMG guidelines** | **Final classification** | **Clinvar ID/ Novel** |
| --- | --- | --- | --- | --- | --- | --- | --- | --- | --- |
| RP 7 | *LZTR1* (NM_006767.4) | c.824G>A | 9 | Missense | p.Arg275Gln | PM2(supporting)/PP2(supporting)/BP4(supporting) | 1points=2P-1B | VUS_LP | Novel ([SUB15708030](https://submit.ncbi.nlm.nih.gov/subs/clinvar_wizard/SUB15708030)) |
| RP27 | *MAPK1 (*NM_002745.5) | c.128A>T | 2 | missense | p.Tyr43Phe | PM1(supporting)/PM2(supporting), BP4(moderate) | 0points =2P-2B | VUS | Novel ([SUB15708036](https://submit.ncbi.nlm.nih.gov/subs/clinvar_wizard/SUB15708036)) |

**Supplementary Table 2. Exploratory DGIdb analysis of drug–gene interactions**

This table summarises the top-ranking interactions between RASopathy-associated genes identified in our cohort and FDA-approved or investigational drugs listed in the Drug–Gene Interaction database (DGIdb v5.0.9). The data are intended as hypothesis-generating and highlight potential avenues for future functional or translational research. They should not be interpreted as direct clinical recommendations, as therapeutic efficacy was not tested in this study.

| **Gene** | **Drug** | **Regulatory Approval** | **Indication** | **Interaction Score** |
| --- | --- | --- | --- | --- |
| BRAF | DABRAFENIB | Approved | antineoplastic agent | 2.474847 |
| BRAF | VEMURAFENIB | Approved | antineoplastic agent | 1.587278 |
| BRAF | ENCORAFENIB | Approved |  | 1.537112 |
| BRAF | TRAMETINIB DIMETHYL SULFOXIDE | Approved | antineoplastic agent | 1.488364 |
| BRAF | PANITUMUMAB | Approved | antineoplastic agent | 1.305095 |
| PTPN11 | SUCCIMER | Approved |  | 1.065384 |
| BRAF | CETUXIMAB | Approved | antineoplastic agent | 0.902985 |
| RIT1 | SELUMETINIB | Approved |  | 0.790967 |
| RAF1 | TOVORAFENIB | Approved |  | 0.669279 |
| BRAF | BINIMETINIB | Approved | antineoplastic agent, DMARD, anti-inflammatory agent | 0.587293 |
| BRAF | COBIMETINIB | Approved | antineoplastic agent | 0.453349 |
| HRAS | SOTORASIB | Approved |  | 0.419308 |
| NRAS | SOTORASIB | Approved |  | 0.395483 |
| BRAF | TOVORAFENIB | Approved |  | 0.391528 |
| NRAS | BINIMETINIB | Approved | antineoplastic agent, DMARD, anti-inflammatory agent | 0.375709 |
| NRAS | TRAMETINIB DIMETHYL SULFOXIDE | Approved | antineoplastic agent | 0.37024 |
| NRAS | ENCORAFENIB | Approved |  | 0.263656 |
| NRAS | PANITUMUMAB | Approved | antineoplastic agent | 0.23729 |
| BRAF | SELUMETINIB | Approved |  | 0.22938 |
| RAF1 | ENCORAFENIB | Approved |  | 0.223093 |
| NRAS | VEMURAFENIB | Approved | antineoplastic agent | 0.203086 |
| NRAS | TORIPALIMAB-TPZI | Approved |  | 0.197742 |
| PTPN11 | AZACITIDINE | Approved | antineoplastic agent | 0.186442 |
| RAF1 | REGORAFENIB | Approved | antineoplastic agent | 0.182531 |
| NRAS | CETUXIMAB | Approved | antineoplastic agent | 0.181709 |
