# Supplementary figures and images for: Germline activating sequence variations in RASopathy spectrum genes: genotype–phenotype correlation in a North Indian cohort
Source: Front Genet. 2025 Nov 11;16:1677143. doi: 10.3389/fgene.2025.1677143 (PMC12643386; doi:10.3389/fgene.2025.1677143)

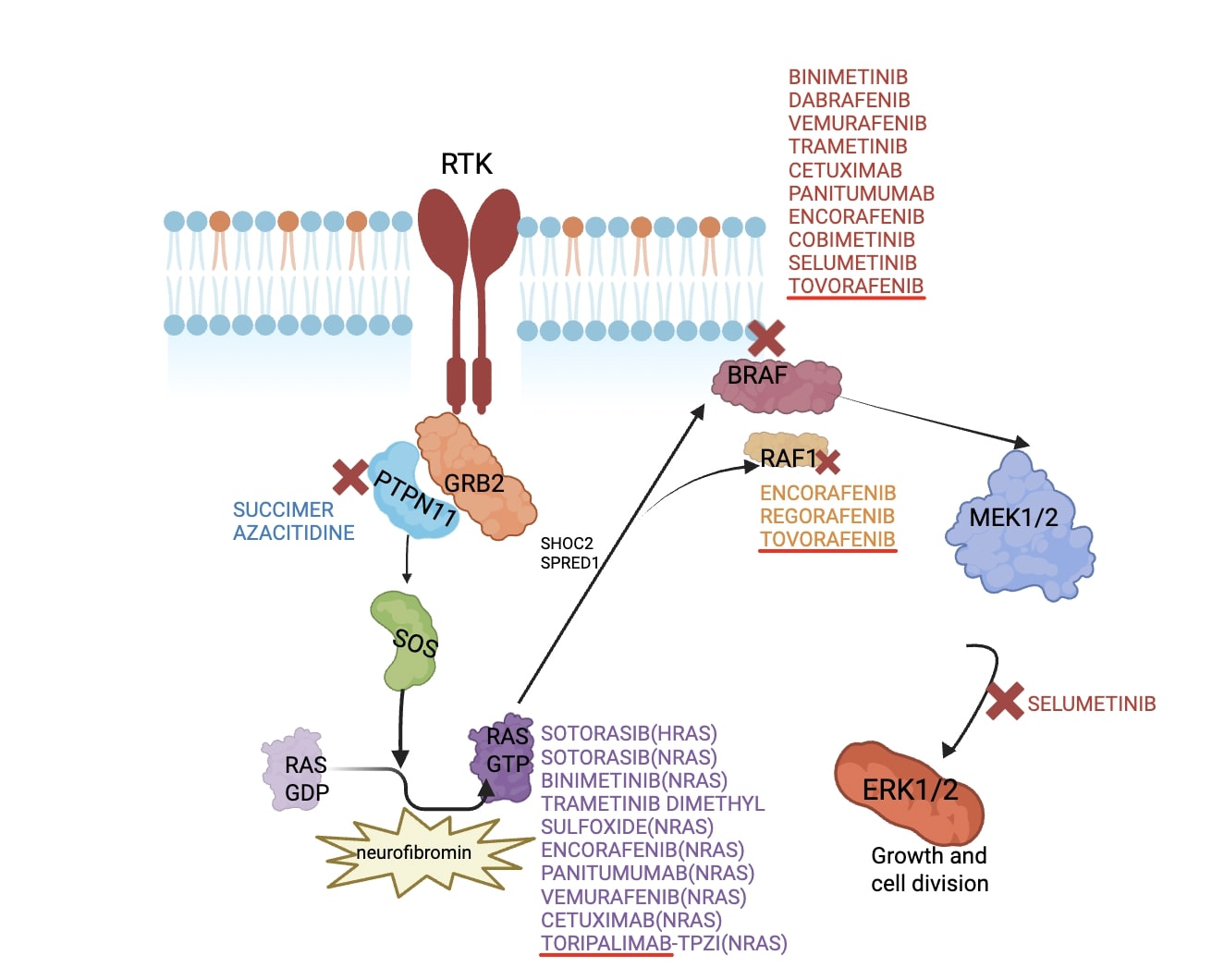

Supplement: Supplementary file 2 [file Image1.jpeg]
